# Visual and Personalized Quality of Life Assessment App for People With Severe Mental Health Problems: Qualitative Evaluation

**DOI:** 10.2196/19593

**Published:** 2020-12-03

**Authors:** David Buitenweg, Dike van de Mheen, Jean-Paul Grund, Hans van Oers, Chijs van Nieuwenhuizen

**Affiliations:** 1 Tranzo Scientific Center for Care and Wellbeing Tilburg School of Social and Behavioral Sciences Tilburg University Tilburg Netherlands; 2 Geestelijke Gezondheids Zorg Eindhoven Institute for Mental Health Care Eindhoven Netherlands; 3 CVO Addiction Research Centre Utrecht Netherlands

**Keywords:** quality of life, qualitative evaluation, visual assessment, e-mental health, assessment app

## Abstract

**Background:**

QoL-ME is a digital visual personalized quality of life assessment app for people with severe mental health problems. Research reveals that e-mental health apps frequently suffer from low engagement and fall short of expectations regarding their impact on patients’ daily lives. Studies often indicate that e-mental health apps ought to respect the needs and preferences of end users to achieve optimal user engagement.

**Objective:**

The aim of this study was to explore the experiences of users regarding the usability and functionality of QoL-ME and whether the app is actionable and beneficial for patients.

**Methods:**

End users (n=8) of QoL-ME contributed to semistructured interviews. An interview guide was used to direct the interviews. All interviews were audiorecorded and transcribed verbatim. Transcriptions were analyzed and coded thematically.

**Results:**

Analysis revealed 3 main themes: (1) benefit, (2) actionability, and (3) characteristics of the QoL-ME. The first theme reveals that the QoL-ME app was beneficial for the majority of respondents, primarily by prompting them to reflect on their quality of life. The current version is not yet actionable; the actionability of the QoL-ME app may be improved by enabling users to view their scores over time and by supplying practical advice for quality of life improvements. Overall, participants had positive experiences with the usability, design, and content of the app.

**Conclusions:**

The QoL-ME app can be beneficial to users as it provides them with insight into their quality of life and elicits reflection. Incorporating more functionalities that facilitate self-management, such as advice and strategies for improving areas that are lacking, will likely make the app actionable. Patients positively regarded the usability, design, and contents of the QoL-ME app.

## Introduction

Quality of life assessment in people with severe mental health problems faces several challenges. First, respondents may not have had the opportunity to develop the abilities necessary to engage in traditional language-based quality of life assessments [1-3]. Alternatively, comorbid intellectual disabilities [3-5] or psychopathology [6-8] may compromise the validity of quality of life results. Second, in mental health, quality of life is understood as an inherently subjective concept that is shaped by individuals’ values and preferences [9-11]. Research underlines this notion [12-14], which calls for further personalization of quality of life measurements. Third, quality of life assessment instruments may promote patient empowerment by providing patients with insight into their quality of life scores, which is an important prerequisite for shared decision making [15-17]. Both patient empowerment and shared decision making have become important goals in mental health services [18,19]. To meet these 3 challenges, an innovative personalized visual quality of life assessment app was developed called *QoL-ME* [20]. The QoL-ME app consists of a core version that can be supplemented with additional modules. The core version involves a mandatory set of 3 universal quality of life domains. In addition, respondents can choose from 8 additional modules. Every module involves a domain of quality of life that respondents may select if it is important for their quality of life. Respondents only answer questions on their selection of additional modules. After filling out the questions, respondents receive direct feedback from the app in the form of an overview of their answers. The QoL-ME app was developed cocreatively in close collaboration with patients, family members, and care professionals [20,21].

Both research and practice reveal that e-mental health apps frequently suffer from low engagement and fall short of expectations regarding their impact on the daily lives of patients. [22-26]. Researchers have, therefore, investigated what factors enable e-mental health apps to bridge the gap from development to high engagement and practical use by patients [27-29]. Generally, these studies [27-30] often indicate that e-mental health apps ought to respect the needs and preferences of patients to achieve optimal user engagement, and 2 specific factors are of special importance. First, it is essential that the app is actionable. An app is actionable if provides a useful base for practical action for patients [31]. Examples of practical action include patients altering their sleep schedule after using an app that has sleep tracking functionality [25] or opting not to engage in a romantic relationship based on the results of a self-management app [26]. Second, use of the app ought to be beneficial to patients. An app should effectively address an issue patients care about so that they derive a tangible benefit from utilizing the app [31].

End users played a vital role in the development of the QoL-ME app. In the context of this development, participants rated the usability of the app as “very high [20].” It is unknown, however, whether the intensive user-involvement and positive usability rating translate to an instrument that is of use to patients in real-life settings.

In light of the discrepancy between the potential of e-mental health apps and their lack of impact on patients’ daily lives, it is crucial to investigate the experiences of patients who used the QoL-ME app. In addition, it is of special importance to examine to what degree the QoL-ME app is actionable and beneficial to its users. The aim of this study was to explore the experiences of users regarding usability and functionality and whether the app was actionable and beneficial for patients. To this end, participants who had used the QoL-ME app were interviewed.

## Methods

### Participants

This study included 3 specific populations of people with severe mental health problems: people with psychiatric problems, people being treated in forensic psychiatry, and people who were experiencing homelessness. Individuals experiencing homelessness were included in this study because of the high prevalence of severe mental health problems in this group [3,32,33]. These groups may have difficulties with traditional language-based quality of life assessments because of fewer educational opportunities [1-3], co-occurring intellectual disabilities [3-5], and compromising psychopathology [6,7]. A consortium consisting of 6 societal institutions was formed to facilitate this study and the broader research project. These institutions included a multimodal day treatment center for multiproblem young adults, a hospital for forensic psychiatry, a mental health institution, a day center for people who are homeless, and 2 research institutions focusing on lifestyle, homelessness, and addiction. Participants were recruited with the help of the consortium partners.

The sample consisted of individuals who had gained experience with the QoL-ME app in the context of a psychometric evaluation of the app. In this psychometric evaluation, respondents were invited to use QoL-ME monthly for a period of 6 months. A specific inclusion criterion of at least 5 uses of QoL-ME was employed. This criterion ensured that patients had sufficient experience with QoL-ME to be able to contribute valuable information. The aim was to include enough participants to reach saturation in the sample, defined as a lack of new information in the final 2 interviews [34].

Ethical approval was obtained from the Ethics Committee of the Tilburg School of Behavioural and Social Sciences at Tilburg University (EC-2015.44). Informed consent was obtained from each participant. All procedures performed in this study involving human participants were in accordance with the ethical standards of the institutional and national research committee and with the 1964 Helsinki Declaration and its later amendments or comparable ethical standards.

### The QoL-ME App

A group of 59 patients contributed to the development of the QoL-ME app. The iterative development comprised 6 iterations divided over 3 stages. In the first stage, patients were invited to share their ideas regarding design and functionality. In the second stage, initial designs and wireframes were developed into a fully functioning prototype. This process was guided by the feedback and ideas of patients. The prototype was subjected to a usability evaluation in the final stage [20].

QoL-ME encompasses 2 separate core versions. The first core version targets people with psychiatric problems and people treated in forensic psychiatry and includes 3 domains of the Lancashire Quality of Life Profile (LQoLP) [11]: safety, living situation, and finances. A recent study indicates that these 3 LQoLP domains are universal [12]. The LQoLP uses a 7-point Likert scale, ranging from 1 (cannot be worse) to 7 (cannot be better). The second core version is tailored to people who are homeless and comprises the Dutch version of the Meaning in Life Questionnaire (MLQ) [35], a 10-item measure that assesses both the presence of meaning in one’s life and the search for meaning in life. Research indicates that having meaning in life is especially important for people who are homeless [36,37]. The MLQ also uses a 7-point Likert scale, ranging from 1 (completely disagree) to 7 (completely agree).

The additional modules served to ensure the personalization of the QoL-ME app. The following 8 domains of quality of life were included: (1) support and attention, (2) social contacts, (3) happiness and love, (4) relaxation and harmony, (5) leisure, (6) lifestyle, (7) finances, and (8) health and living. These domains were identified in a visual concept mapping study of the quality of life of people with severe mental health problems [21].

**Figure 1:**
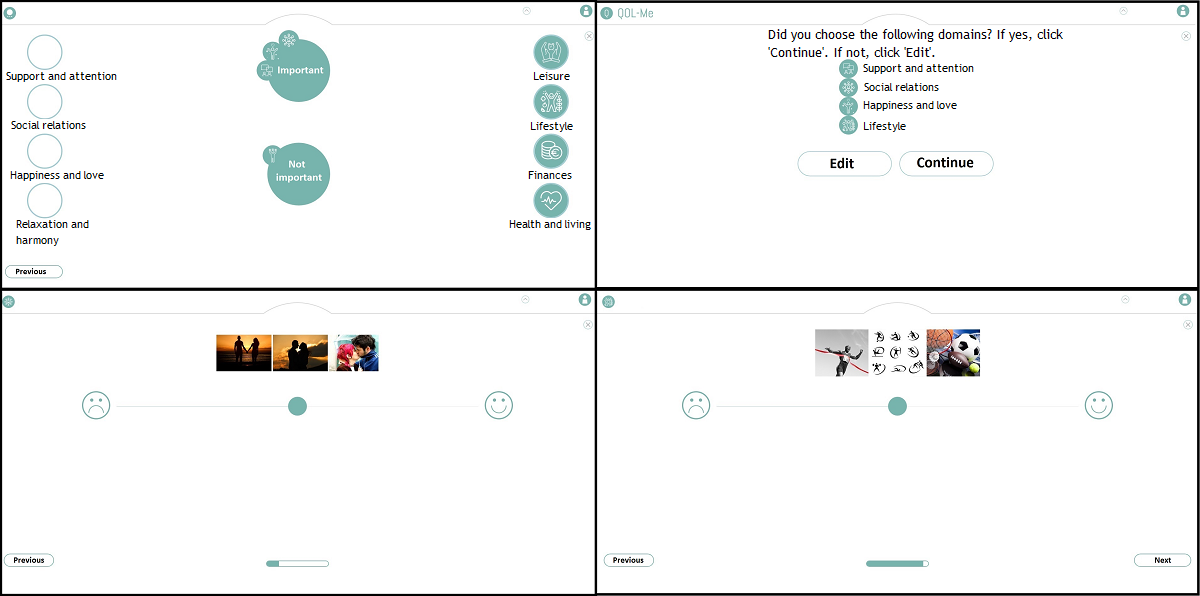
Four screenshots depicting the additional modules of the QoL-ME. The top-left panel displays how respondents select additional modules. Respondents are invited to drag eight icons, corresponding to the eight modules, to a circle that says ‘important’ or a circle that says ‘not important’. The top-right panel shows how respondents are asked to confirm their choice of additional modules. The two bottom panels provide examples of items of the additional modules.

Domains are assessed using 2 to 4 visual items. Every visual item contains 3 pictures that together denote an aspect of quality of life. Users respond to these items using a visual analog scale with visual anchors. Figure 1 depicts how respondents select additional modules and provides 2 examples of items in the additional modules.

In the QoL-ME app, users first indicate which of the 2 core versions is appropriate for them and respond to the items of that core version. Next, they select a combination of the 8 additional modules based on their importance. Upon completing the visual items of the additional modules, users are provided with an overview of their answers.

A thorough description of the development of the QoL-ME app, including additional visual material, is provided elsewhere [21].

### Approach

A qualitative research approach was employed to explore the participants’ experiences with the QoL-ME app. Specifically, individual semistructured interviews were utilized as they allowed participants to elaborate on their experiences and allowed the researcher to clarify any confusing or unclear questions when necessary. In addition, the context of individual interviews enabled reference to the QoL-ME app to make questions more tangible. The use of semistructured interviews combined a guiding structure providing participants freedom to expand on their answers.

### Content of the Interview

An interview guide was used in this study. Four sources of information were consulted to inform this interview guide (Table 1). First, insights regarding patients’ needs and preferences concerning QoL-ME gained during development were fed back into the interview guide. Second, the Health Information Technology Acceptance Model (HITAM) was consulted [38]. The HITAM describes consumers’ behavioral intentions toward the use of health technology. Third, relevant information was extracted from 2 questionnaires designed to evaluate mobile health apps—the Mobile App Rating Scale (MARS) [39], and the App Chronic Disease Checklist (ACDC) [40]. Fourth, scientific literature was examined, and information regarding patients’ needs and preferences regarding mobile mental health apps was extracted [27,31,41,42]. The 18 topics were grouped into 5 overarching themes (see Table 1), and each theme was introduced using a short primer.

**Table 1:** Overview of the interview guide used in this study. The guide includes the different factors queried in this study, their origin, and the questions used to explore them.

Topic		Question		Source	
**Deriving value**					
	Beneficial		Did using the QoL-ME benefit you? And if so, how? If not, what changes can we make for you to derive benefit from using the QoL-ME?		Development, HITAM^a^, [31]
	Actionable		Did your use of the QoL-ME result in actions? If yes, which actions?		
**Content and results**					
	Number of questions		What do you think about the number of questions in the QoL-ME?		Development
	Match questions and respondents		To what degree did the questions of the QoL-ME match your world and experiences?		
	Feedback		At the end of the QoL-ME, you can review your answers. What do you think about that?		Development, ACDC^b^
	Comparing results		Would you welcome the possibility to compare your own results with others and why?		Development
	Stimulation / motivation		What do you think about the possibility to stimulate the use of an app such as the QoL-ME through push messages or other mechanisms?		Development, ACDC
**Usability**					
	General usability		What do you think about the QoL-ME’s usability? Are there any changes we can make to improve its usability? If yes, which changes?		Development, HITAM, ACDC, [31,41]
	Structure		Does the QoL-ME have a clear structure according to you? Why/why not?		Development, ACDC, MARS^c^
	Intuitive design		Did you have to learn or practice before using the QoL-ME? If yes, what did you have to learn or practice?		ACDC, MARS
	Appearance		What do you think about the appearance of the QoL-ME?		MARS
	Performance		Did you run into any problems using the QoL-ME on your phone/tablet/laptop/computer? If yes, which problems?		ACDC, MARS
	Barriers		Were you unable to use the app for any reason? If yes, what reasons?		HITAM
**Personalization**					
	Personalized content		What did you think about selecting your own topics in the QoL-ME?		Development, ACDC, MARS
	Personalized appearance		During the development of the QoL-ME, some participants indicated a preference for customizing the appearance of the QoL-ME. What do you think about that?		Development, ACDC, MARS, [27]
**Trust**					
	Privacy/data security		Do you think that your data is safe and confidential in the QoL-ME? Why?		Development, ACDC, [42]
	Transparency		Do you know which parties get to see your data and what they do with them?		Development, [27]
	Professional credibility		What do you think about the credibility of the QoL-ME?		ACDC, MARS

^a^HITAM: Health Information Technology Acceptance Model.

^b^ACDC: App Chronic Disease Checklist.

^c^MARS: Mobile App Rating Scale.

### Data Analysis

A deductive, or theoretical [43], analysis approach was employed, starting from a specific predefined research question. All interviews were audiorecorded. The recordings were transcribed verbatim, and transcripts were coded thematically utilizing the 6-step method outlined by Braun and Clarke [43] in order to capture user experience themes. Initial themes were continuously refined and reflected on using a deductive approach. In step 1, the researchers familiarized themselves with the data through checking and verifying the accuracy of the transcripts. Step 2 involved the selection of an initial set of codes and themes based on the first 3 interviews. Codes were used to label and organize qualitative data. Codes with similar content were clustered into overarching themes. The coding was performed using ATLAS.ti (version 8, ATLAS.ti Scientific Software Development GmbH). The 2 researchers compared their initial codes to ensure consistency throughout the coding process. Once the initial set of codes was confirmed, the researchers independently coded all of the interviews using the initial set. This set was modified or added to if necessary. Once all the interviews had been coded and the researchers reached consensus regarding the coding of the transcribed interviews, step 3 involved clustering of the codes into overarching themes. Themes were identified based on recurring codes. In step 4, the researchers discussed the themes and modified them, when required, to reach consensus on content and labeling. Step 5 encompassed interpreting and naming emerging themes. The results of the 6-step analysis method were reported in step 6 [43].

### Procedure

Participants who contributed to the quantitative evaluation of the QoL-ME (DC Buitenweg, et al, unpublished data, 2020) were invited to participate in the interview. Participants who met the inclusion criteria were contacted via email, via care professionals at the consortium institutions, or via telephone if possible. Participants who expressed interest in contributing were provided with additional information on the qualitative study. Once a participant agreed to contribute, the researcher (DB) and participant scheduled an appointment for an interview. Interviews were held at the institution that supported the participant, or at a neutral location such as a café. Prior to the interview, the researcher provided a detailed explanation of the study and of what was expected of the participant. Moreover, the researcher explained that there were no right or wrong answers and that it was important that participants freely shared their opinions. Next, the researcher and participant went through the QoL-ME together to ensure that all participants had a refreshed understanding of the QoL-ME app. The interview guide (Table 1) steered the interview, while the interviewer elaborated on topics when necessary. Upon completing the interview, the interviewer explained how the data would be analyzed and how this aided the study. Participants were given time to ask any further questions. The interview ended when all questions were addressed whereupon the participant received a gift voucher. The duration of the interviews varied between 17 and 42 minutes and the average duration was 31 minutes.

## Results

### Participants

A group of 19 patients contributed to at least 5 assessments in the psychometric evaluation of the QoL-ME app. Of these 19 patients, 10 patients initially agreed to participate in an interview. The 9 patients who declined reported a lack of time or interest as their reason for declining to participate in the interviews. Of the 10 patients who initially agreed, one patient could no longer be reached and another was too busy to schedule an appointment. Therefore, 8 individuals with severe mental health problems participated in this study. We were unable to continue including participants until saturation because the number of experienced users who agreed to participate in the interviews was relatively low. Participants’ demographic characteristics are provided in Table 2. Five participants were male, the mean age of participants was 34 (SD 12 years), and 5 of the 8 participants had a Dutch cultural background. All participants had experienced using QoL-ME by contributing to the psychometric evaluation of QoL-ME. On average, participants had filled out QoL-ME 6 times (range 5-7) over a period between 4 and 6 months. Of 8 participants, 6 reported using QoL-ME on their personal smartphone, and the remaining 2 participants used their personal computer. Participants primarily used QoL-ME at home, while some reported using QoL-ME at their care institution.

**Table 2:** Demographic characteristics of the participants.

Participant	Age (years)	Gender	Cultural background	Level of education	Occupational status
1	18	Male	Dutch	Basic	Paid employment
2	41	Male	Turkish	Basic	Volunteer work
3	39	Female	Dutch Antilles	Basic	Education
4	33	Male	Dutch	Basic	Unemployed
5	43	Female	Dutch	Basic	Volunteer work
6	27	Female	Dutch	Intermediate	Unemployed
7	52	Male	Dutch	Intermediate	Volunteer work
8	19	Male	Indonesian	Basic	Unemployed

### Main Findings

The following 3 themes were identified based on analysis of the interviews: (1) benefit, (2) actionability, and (3) characteristics of QoL-ME. An overview of the codes and themes is provided in Multimedia Appendix 1 and includes both an overview in table form and a graphical depiction of the network of codes and themes. As the first 2 themes pertain to the 2 concepts (beneficial and actionable) that were of special interest in this study, these themes are discussed in more detail.

### Benefit

According to 6 of the 8 interviewees, using QoL-ME was beneficial to them. All 6 of these participants mentioned that using the app made them more aware of their level of satisfaction on the life domains incorporated in the QoL-ME app.

Well, because of the questions that are asked, you start to think about what you do and don't have. In principle, I am actually satisfied with everything. But you are going to look at how you are doing. In your relationships, your family and your finances.

Participant 6

For some participants, being confronted with their dissatisfaction on some domains drove them to look for ways to improve their situation.

The questions about income and whether you were satisfied with how much money you can spend made me think. When I have a job later on, I have more room for big expenses. So I started thinking about that. Yeah, that’s it, yes.

Participant 7

For other participants, the QoL-ME app facilitated the realization that they were happier than they thought they were.

Ehmm. I started to think more consciously about how happy I actually was. And I turned out to be happier than I actually thought.

Participant 8

The 2 participants for whom the QoL-ME app was not beneficial mentioned already having sufficient insight into how satisfied they were with their lives as the main reason for this lack of benefit:

No, no the questions that were asked, I already had some kind of insight in them. In those areas. So no I didn't really get anything out of it.

Participant 5

Both participants did feel that the QoL-ME app would be more beneficial to them if they lacked this insight:

[Interviewer:] And if you hadn’t known how you were doing in life?

[Participant:] Yes, if you don’t have that then you can discuss it with someone: oh, this is not going well so maybe I should do something with that. So then it would help.

Participant 1

### Actionability

For 3 participants, the QoL-ME app proved to provide a useful base for taking actions in their daily lives. One participant mentioned that using the QoL-ME app assisted her in maintaining of social relationships.

Well, for example I had not seen someone for a long time and I thought: let me call them. I tried to make contact. And you are also busy with your own life, I know, but I did think about that.

Participant 3

Another participant spoke of being more careful in public transportation as a consequence of filling out the “Safety” domain:

[Interviewer:] And based on that, have you done something, changed something to what you normally do? For example in the area of personal safety?

[Participant:] Yes, subconsciously I did, because if I don't feel safe and I don't have to leave, then I stay inside. And for example if I travel by public transport and I see something strange then I get off. You start thinking more about these things.

Participant 2

None of the participants reported discussing their QoL-ME results with others, but 2 participants acknowledged the possibility:

Then you have it right in front of you: things are not going so well. And then you can discuss that with someone. Okay, how are we going to improve this?

Participant 1

Five participants reported not having taken any concrete action based on their experiences with QoL-ME. Two participants indicated that incorporating the option to compare current results with previous results would improve the actionability of QoL-ME.

[Participant:] what seems interesting to me is to see if your answers change over the different measurement moments.

[Interviewer:] Why is that interesting to you?

[Participant:] o see if it changes or if I am consistent. Because every day is different.

[Interviewer:] Yes, and if you could see that change, how would that affect how the App benefits you?

[Participant:] When I see that I am very satisfied with a certain topic one day and not at all the next, then I start to think ‘hmm, what is the reason for that?’ Where does that difference come from? And then it is also easier to do something with it.

Participant 4

Regarding the potential negative effects of confronting users of the QoL-ME app with a decrease in their quality of life scores in the absence of care professionals, none of the participants expected this to be a problem.

Yes for some people you wouldn't want to see that of course. But I feel like ... it's how you feel at the time. The situation may still be the same, but the way you deal with it may be different. You can feel different every day.

Participant 3

Some participants provided tips for improving the clarity of the results section, which would also improve the actionability of QoL-ME but this is discussed under the third theme. One participant recommended including advice for how to improve low QoL-ME scores to improve its actionability. He used a food diary app as an example. Users register what they eat on a daily basis and the app generates an advice based on user input.

[Participant:] Yes, okay, so it really is for you… yes maybe you can generate an advice at the end of such a test. We see from your answers that you score negative on these topics and maybe you can think about that. Something like that.

[Interviewer:] Is that also a way to get more benefit from it?

[Participant:] Sure, I think so. That is ultimately what you want, a system that thinks along with you. I have an example, a silly example maybe, but I have an App from the nutrition center. This keeps track of exactly what you eat, and there is also advice. We see that you eat too much salt and too many unhealthy products. And then you are really triggered like I have to fall within the margins of that App. Or something like a pedometer, things like that.

Participant 4

### Characteristics of QoL-ME

Overall, participants welcomed the opportunity to view their results upon completion. Three participants provided specific advice for improving the clarity of the results section to increase the actionability of QoL-ME:

[Interviewer:] And the results you get to see at the end, did you think they are clearly displayed?

[Participant:] Ehm, I think in the second part, that you could add something like a number or something, I think.

[Interviewer:] Add a number or replace something with a number?

[Participant:] Add a number. So that you can see more clearly what it is ... or a percentage or something I am not sure. At least something that reflects it more clearly.

Participant 1

Seven participants appreciated the possibility to personalize the content of QoL-ME. The one participant who disagreed indicated that he found all domains important and therefore preferred a version in which no choices had to be made. Participants were divided regarding the option to personalize the appearance of the QoL-ME app. Four participants welcomed this functionality, but the other participants thought it added too little value.

Several participants commented on the content of the QoL-ME app. One participant thought that the items on the financial situation of respondents were too direct and advised an alternative formulation. Four participants commented on the images used in the additional modules of the QoL-ME app. One participant recommended more variety (ie, avoiding the use of similar pictures). Three participants reported that some of the images used were unclear to them. They advised including a written description of the content of the item using a word or a short sentence for clarification.

None of the participants had trouble with the duration of filling out or the number of questions. Three participants did miss a clear ending message, and they advised including this. One participant had issues with the low contrast between foreground and background elements due to her visual impairment. Seven participants thought the QoL-ME app looked professional, primarily due to its uncluttered and simple layout.

No participants reported having insight into which persons and parties had access to their data. Still, 6 participants trusted the security of their data. The inclusion of a disclaimer containing information regarding data access and use was a welcome addition for 7 participants.

In general, all participants were very positive regarding the design and usability of the QoL-ME app. Participants appreciated the clear structure of the app and favored the navigational system.

## Discussion

### Principal Findings

This study explored the experiences of users regarding the usability and functionality of the QoL-ME app and whether the app was actionable and beneficial for patients. As it is important that an e-mental health tool such as the QoL-ME app is both beneficial and actionable to its users, special attention was paid to these concepts. The interviews revealed that using the QoL-ME app is beneficial to most users, primarily by pushing them to consider their satisfaction in various life domains. The QoL-ME app did not prove to be actionable for most respondents. In addition, respondents were positive about the design and usability of the QoL-ME app but also had some tangible tips and advice for improvement.

The main way in which the QoL-ME app was beneficial to users was through providing insight and facilitating reflection. Some respondents indicated that their use of the QoL-ME app made them realize that they were more satisfied with their lives than they had expected. This result echoed findings by Morton and colleagues [26] in their evaluation of a quality of life self-monitoring tool for people with bipolar disorder. Respondents also indicated that they were sometimes surprised by how high their scores were, which led to the insight that “things were not so bad.” Two participants indicated that they already had sufficient insight into their own quality of life and therefore derived no extra benefit from using the QoL-ME app. This finding echoed results found by Berry and colleagues [44], who investigated views on using digital self-management tools among people with severe mental health problems; a number of participants who contributed to that qualitative interview study indicated that they were already sufficiently self-aware and expected little benefit from using digital self-management tools [44].

Participants provided 3 useful suggestions for making the QoL-ME app more actionable. First, half of the participants proposed including numerical indicators of users’ satisfaction scores for every item or domain. The results section of the current version of the QoL-ME app does not include numbers but only shows a bar that is partly filled based on underlying scores. The Personal Health Information Self-Quantification System model [45] outlines how self-quantification is of vital importance for the self-management of health. In the model, self-quantification is described as the step in which an individuals’ goal (having a good quality of life) is transformed into objectively measured units [45]. Results from Morton and colleagues [26] confirm the importance of quantification, as respondents indicated that it was the quantification of their quality of life that enabled self-management. A second important suggestion for make the QoL-ME app more actionable, from 2 participants, was to incorporate practical advice for improving users’ satisfaction on certain life domains. The tool evaluated by Morton and colleagues [26] was integrated in a larger digital self-management platform that included practical advice and strategies for self-management. The results section of the tool provided direct links to these strategies, a feature that participants were very enthusiastic about [26]. Expanding the QoL-ME app to include similar functionality will likely make the app more actionable for users. The third suggestion pertained to enabling users to follow the development of their quality of life scores over time. Every participant saw this as a welcome addition. This finding was in accordance with those of Morton and colleagues [26] and Berry and colleagues [44]. These 3 suggestions may be used to strongly improve how beneficial and actionable an assessment tool such as the QoL-ME app is to patients.

Several participants acknowledged the possibility of discussing the results of the QoL-ME app with other individuals such as a family member or professional caregiver. The fact that none of them did so may be an indication of social isolation, which has frequently been reported in this population [1-3]. Moving toward self-management, future versions of the QoL-ME app may actively encourage users to share their results and include practical suggestions for decreasing social isolation.

Participants were unanimously positive regarding QoL-ME’s usability. They found the application easy to use, appreciated its linear structure and prized the calm and clean layout. These results confirm the findings from the usability evaluation that made up the last part of the development of QoL-ME [20] and serve as additional corroboration of the design recommendations [41,46] consulted during the apps’ development. Several respondents preferred combining the visual material used in the additional modules with a word or short sentence to denote the content of its item. Comparable pictorial assessment instruments, such as the pictorial version of the Aachen Quality of Life Interview [47] and the pictorial motivation scale in physical activity [48] also combine both visual and verbal content. Respondents had very limited insight into which persons and parties had access to their data. This did not deter them from engaging with QoL-ME. This may be because respondents used QoL-ME in the context of a scientific study or because participating did not require respondents to share any personal information.

The results draw attention to several ways in which the QoL-ME app may be modified so that it is more beneficial for patients. Future research may further investigate what images used in the QoL-ME app are unclear and identify alternative images. Moreover, the results section of the app may be updated to display the development of results over time. In addition, following the example by Morton and colleagues [26], QoL-ME may be integrated into a larger self-management platform for people with severe mental health problems.

### Strengths and Limitations

This study provides an important contribution to the field of e-mental health app development. The qualitative methodology provided patients with the opportunity to share their opinions regarding the usability and functionality of QoL-ME and to what degree the app was beneficial and actionable to them. The results draw attention to the fact that patients require functionalities that target their needs before an app becomes beneficial to them. Specifically, patients require functionality targeting self-management. In addition, the content of the interview was partially derived from existing frameworks that have proven to be effective for evaluating health apps [49].

Still, the results do need to be interpreted in light of 3 limitations involving the sample of participants who contributed to this study. The first limitation pertains to the size of the convenience sample used in this study. The eligible research population, based on the criterion of having completed at least 5 measurements, was small. Still, the results provide important insights into user experiences and the extent to which the QoL-ME app was beneficial and actionable for users. Once a larger group of patients starts using QoL-ME, additional research will have to reveal whether these results extend to the larger population. Analyses revealed that saturation, defined as a lack of new information in the final interviews, was not attained in the sample. The final 2 interviews did contain new information, but these were not substantial insights and no changes to the codes or themes were made based on these interviews.

The context in which participants gained experience with QoL-ME formed a second limitation. Participants were aware that they had used the QoL-ME in the context of a scientific study in which the psychometric quality of the QoL-ME was evaluated. Moreover, participants were incentivized to use the QoL-ME and to participate in the interviews. Therefore, their use of the QoL-ME may not represent use in a real-life setting, and their responses in the interviews may have been biased. To counter possible bias due to the incentives, the researcher indicated that respondents were allowed to freely give their opinions before the interviews started. Future research may investigate to what degree future results are consistent this study's results when patients’ who use QoL-ME on their own accord are interviewed.

The third limitation pertains to the absence of data on participants’ medical background, such as psychiatric diagnoses or symptom severity. Still, all participants received care from the consortium institutions, and we can therefore be certain that they were part of the target population. Future research may investigate whether individuals with specific symptoms or diagnoses have differing experiences using QoL-ME.

### Conclusions

The QoL-ME app can be beneficial to users as it provides them with helpful insight into their quality of life. Including added functionality in support of self-management, such as advice and potential strategies for improving quality of life domains with which app users are dissatisfied will likely make the QoL-ME app more actionable. Overall, the patients who were interviewed positively regarded the usability, functionality, and contents of the QoL-ME app.

## Appendix

Multimedia Appendix 1 Overview of codes and themes identified in the qualitative analysis.

## Abbreviations

ACDC: App Chronic Disease Checklist
HITAM: Health Information Technology Acceptance Model
LQoLP: Lancashire Quality of Life Profile
MARS: Mobile App Rating Scale
MLQ: Meaning in Life Questionnaire
