# Supplementary material for: Visual and Personalized Quality of Life Assessment App for People With Severe Mental Health Problems: Qualitative Evaluation
Source: JMIR Ment Health. 2020 Dec 3;7(12):e19593. doi: 10.2196/19593 (PMC7746488; doi:10.2196/19593)
Supplement: Multimedia Appendix 1 [file mental_v7i12e19593_app1.pdf]

Multimedia Appendix 1. Codes and Themes

To provide additional insight into the codes and themes identified in the qualitative analysis, this Multimedia appendix provides (1) a list of all codes and themes in the form of a table, and (2) an overview of the network of codes and themes.

1. **List of codes and themes**

THEMES	CODES
**ACTIONABILITY**	Actionable - results (positive)
	Actionable- results (negative)
	Actionable- results (neutral)
	Hypothesis - development of scores over time (negative)
	Hypothesis - development of scores over time (positive)
**OBTAINED BENEFIT**	Benefit (negative)
	Benefit (neutral)
	Benefit (positive)
	Characteristics of the QoL-ME - viewing results (improvement)
	Characteristics of the QoL-ME - viewing results (negative)
	Characteristics of the QoL-ME - viewing results (positive)
	Hypothesis - effects of decrease in scores
	Hypothesis - comparing with peers (negative)
	Hypothesis - comparing with peers (positive)
**CHARACTERISTICS OF THE QOL-ME**	*Content*
	Content - images
	Content - improvement
	Content - questions
	Characteristics of the QoL-ME - number of questions (negative)
	Characteristics of the QoL-ME - number of questions (positive)
	Characteristics of the QoL-ME - time to fill out QoL-ME (negative)
	Characteristics of the QoL-ME - time to fill out QoL-ME (positive)
	Hypothesis - length of questionnaire
	Design and usability
	Appearance - credibility
	Appearance - professional
	Hypothesis - design QoL-ME
	Usability - barriers
	Usability - design (improvement)
	Usability - design (negative)
	Usability - design (positive)
	Usability - ease of use
	Usability - general (negative)
	Usability - general (positive)
	Usability - improvement

Multimedia Appendix 1. Codes and Themes

	Usability - login (negative)
	Usability - login (positive)
	Usability - navigation (negative)
	Usability - navigation (positive)
	Usability - navigation (user experiences)
	Usability - structure (negative)
	Usability - structure (positive)
	General experience - area for improvement
	General experience - internal process
	General experience - negative
	General experience - positive
	Device used to fill out QoL-ME
	Hypothesis - general
	Location of filling out QoL-ME
	Personalization
	Personalization - content
	Personalization - design (negative)
	Personalization - design (positive)
	Personalization - general
	Hypothesis - personalizing content
	Hypothesis - pop-up messages (negative)
	Hypothesis - pop-up messages (positive)
	Privacy and data security
	Privacy and data security - general (negative)
	Privacy and data security - general (neutral)
	Privacy and data security - general (positive)
	Privacy and data security - knowledge
	Privacy and data security - knowledge (improvement)

2. Network of codes and themes

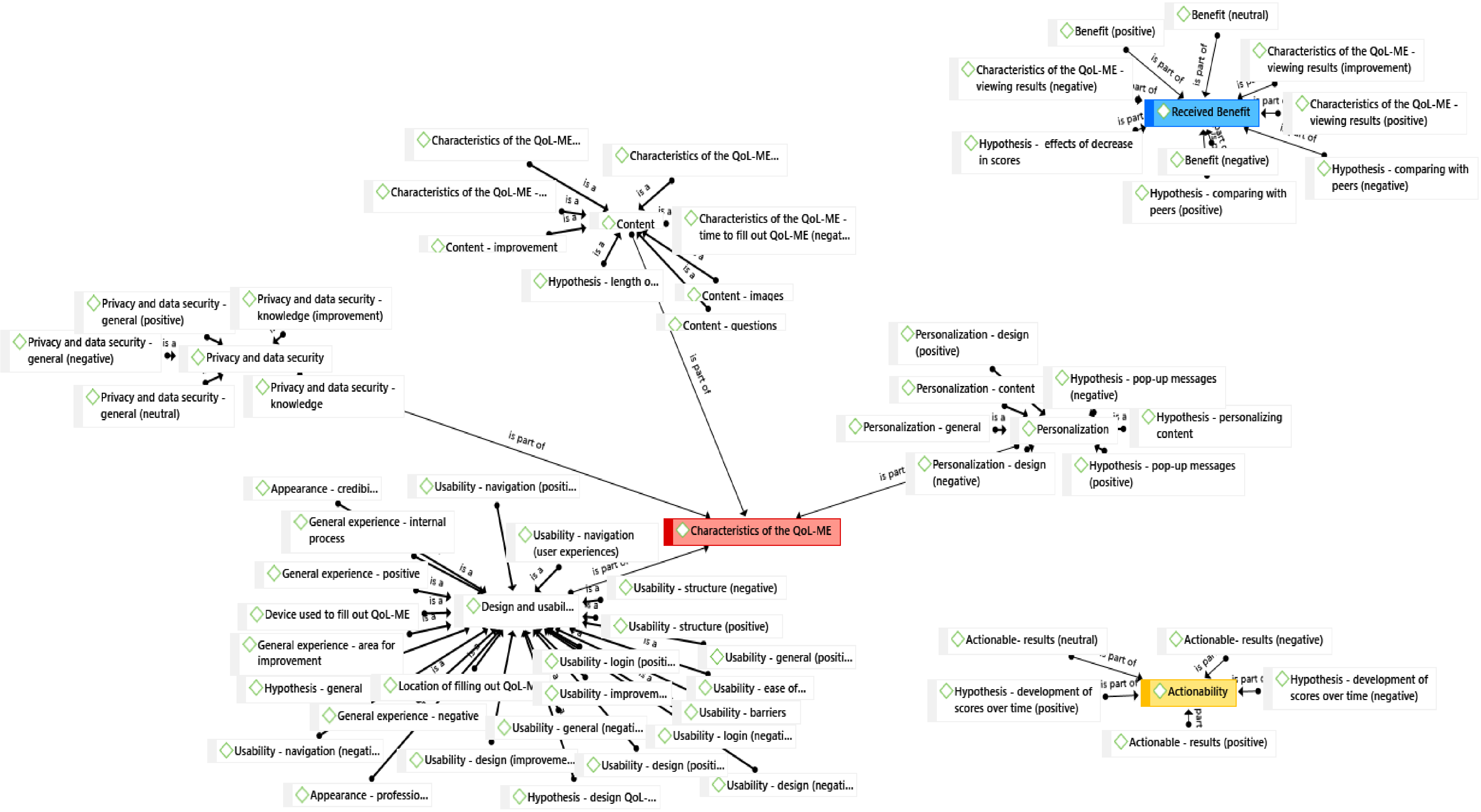

Multimedia Appendix 1. Codes and Themes
